# The state of transience, and its influence on the wish to die of advanced disease patients: insights from a qualitative phenomenological study

**DOI:** 10.1186/s12904-024-01380-z

**Published:** 2024-02-26

**Authors:** Alazne Belar, Maria Arantzamendi, Philip Larkin, Iñaki Saralegui, Yolanda Santesteban, Nerea Alonso, Marina Martínez, Carlos Centeno

**Affiliations:** 1 Institute for Culture and Society, University of NavarraIdiSNA, Pamplona, Spain; 2 Palliative and Supportive care service, University Hospital of Lausanne and University of Lausanne, Lausanne, Switzerland; 3 OSI ARABA, Osakidetza, Vitoria-Gasteiz, Spain; 4 Hospital San Juan de Dios, Obra Social “La Caixa”, Pamplona, Spain; 5 Clínica Universidad de Navarra, IdiSNA, Pamplona, Spain; 6 Institute for Culture and Society, University of Navarra, Clínica Universidad de Navarra, IdiSNA, Pamplona, Spain

**Keywords:** Wish to die, Desire to die, Qualitative research, Phenomenological approach, Living experience

## Abstract

**Background:**

The experience of Wish to Die is common in patients living with Advanced Disease. It has been studied worldwide and qualitative studies have contributed to the understanding of the complexity of the phenomenon of the WTD but a deeper understanding on the individual’s views is still needed. The objective of this study was to identify common characteristics of the experience of wish to die in advanced disease.

**Methods:**

A phenomenological study was carried out with multicenter participation of patients with advanced disease who had expressed their wish to die to health professionals. Semi-structured interviews were employed to obtain an in-depth perspective of each patient’s lived experience. A phenomenological analysis of the data collected was performed to describe and explore the characteristic aspects of the phenomenon under study.

**Results:**

Fourteen patients with advanced disease were interviewed. Most of them had cancer. In the analysis of the patients’ accounts of their experiences, three common characteristics were identified: a) experiencing a state of transience; b) the attempt to reconnect with oneself; and c) additional disease-related aspects that influence the wish to die. Patients expressed the need for a safe space to address the wish to die and the importance of receiving care that considers both ‘being’ and ‘doing’.

**Conclusions:**

Patients with advanced disease and wish to die experience a state of transience where the patient lives and ephemeral state of existence. Interventions focused on reinforcing the intrinsic value of the individual emerge as essential components of a compassionate accompaniment of those facing the wish to die.

**Supplementary Information:**

The online version contains supplementary material available at 10.1186/s12904-024-01380-z.

## Background

The experience of the wish to die (WTD) in patients with advanced disease is a phenomenon that has been studied worldwide. This is not surprising considering that 11% and 55% patients present it occasionally [1–3], while in 8-17% of the cases these feelings or thoughts are more persistent [1, 4, 5]. Furthermore, between 4 and 11% of seriously ill patients experiencing such phenomenon present real or hypothetical intention to hasten death [1, 6]. Therefore, this phenomenon ranges from mere experiences of WTD to the explicit request for euthanasia.

Studies have identified factors associated with the WTD. These factors come from different spheres of the person: the existential sphere (e.g. lack of meaning in life, lack of dignity, demoralization), the physical sphere (e.g. symptom burden, drowsiness), the social sphere (e.g. feeling a sense of shame), or the psychological sphere (e.g. anxiety, depression) [1, 7–12].

Initial qualitative studies have been conducted in Canada, Australia, China and the USA [13]. A recent meta-ethnographic has revealed that, in the last decade, several European investigations have incorporated diverse cultural and social context information, reinforcing the need for a comprehensive view of suffering. Such studies have considered not only physical distress but also psychological, social, or existential aspects as crucial in expressions of the WTD [14] although this is not always the case [15]. Until now, qualitative studies have focused mainly on understanding what the patients want to say and the meaning they attribute to the WTD expressions that they have used.

Within some qualitative studies, the reasons for the WTD may include physical, psychological, social and/or existential aspects [14, 15]. Patients weigh up reasons differently, some of which may be in the present moment while others may refer to hypothetical events in the future. Moreover, it seems that these reasons do not fully explain the WTD [15].

It is necessary to understand what the person wishes for (intention) and the meaning it attributes to these expressions. The intentions reported in the literature include a wide variety of typologies including the wish to live, the acceptance of dying and the will to die [16]. There may even be multiple coexisting wishes [16] that may seem contradictory from the outside, but that may be compatible for the person as they are situated on a larger framework of personal values [17]. Patients have reported that their prioritization of wishes may change over time due to an inner progress or thinking, or to the changing conditions of the disease and/or care [16] which is in line with a dynamic idea of the WTD [17].

There is also a variety of meanings attributed to the WTD expressions such as cry for help, triggering interpersonal interaction, allowing a life-ending process to take its course, to spare others from the burden of oneself, or to move on to another reality (afterlife) [14, 15]. Patients’ expressions of the WTD may even have different functions, either as a means of communicating or as a form of control [14].

A recent study has examined similarities and differences on the reasons, intentions, and the social interactions of the WTD on patients with four different types of diseases: neurological conditions, organ failure, cancer, and frailty due to age [3]. This investigation identified that in all patient groups there were clear moments where a wish to die was triggered by specific conditions and transitional points that affected agency and self-understanding [3]. Furthermore, in the same way that there are commonalities according to different disease trajectories, these may equally be expected to exist among individuals’ lived experiences. In this respect, although a more recent study has studied the lived experience of older people with the WTD [18] this topic has not yet been explored in patients with advanced disease.

Qualitative studies have contributed to the understanding of the complexity of the phenomenon of the WTD by yielding insight into patients’ perspectives, as they consider that the many aspects that make up a particular WTD may be bounded to the patients’ personal experiences, moral values, and preferences. Nevertheless, there is still a need for complementary deeper understanding on the individual’s views [15].

Moreover, some authors suggest that data from other cultural settings could add additional important evidence to understand this complex phenomenon [15]. In the Spanish context, to date, no qualitative study has been carried out to investigate the wish to die; studies have focused on its prevalence, its associated factors, or on the effects of proactively exploring WTD [1, 6, 7, 11, 18].

Therefore, the present study intends to help to address this gap by delving deeper into the common characteristics of the patients’ lived experience and focusing more on understanding the experience itself and not so much on the intentions, reasons, or meanings of it. Furthermore, our intention was to investigate the experience of wish to die in patients with advanced disease within the Spanish context as well as the preferences of Spanish patients on how their professionals should approach the subject in clinical encounters and what they expect from them.

## Methods

A hermeneutic phenomenology was conducted, taking into account Max van Manen approach’s underlying ideas, to comprehensively explore the common characteristics of patients’ lived experiences. Phenomenology, defined as the science of the phenomenon [19] represents both a philosophical attitude and a research perspective that delves into essential questions of the person or individual [19–22]. This method is particularly interested in uncovering the defining nature of lived experiences. Employing a hermeneutic-phenomenological approach, we aim to delve into these shared characteristics by identifying the common thread links all those persons’ lived experiences. Furthermore, we seek to understand how these individuals, shaped by their lived experiences, express their preferences regarding the acknowledgment and addressing of this shared experience.

### Setting and sample

Study participants were recruited and interviews were conducted between February and August 2021 in three hospitals: the Clínica Universidad de Navarra (CUN), the Hospital San Juan de Dios (HSJD), and OSI ARABA. The CUN is a private hospital that treats patients from all over Spain (64% of patients come from other regions), the HSJD is a state-contracted hospital, and the OSI Araba is an integrated, hospital-based, and Primary Care organization of the Basque Health Service-Osakidetza.

Purposive sampling was employed to obtain greater variability in the sample. Study participants met the following inclusion criteria: (a) expression of any type of WTD experience (from a sporadic WTD to a persistent experience, including the intentionality of hasten death or not [1]); (b) advanced, progressive and incurable disease; (c) awareness of having a life-threatening disease; (d) no contraindication from the corresponding physician; (e) consent to be interviewed; (f) in hospital or care setting requiring clinical monitoring or follow up, such as an out-patient clinic or at home; (g) ability to speak Spanish.

Healthcare professionals in the palliative care teams in these hospitals were invited to actively collaborate in the recruitment process, a usual procedure in previous studies on the subject, since the protection of particularly vulnerable patients was considered more important than any possible risk of selection bias [15]. These practitioners identified those patients who might meet the inclusion criteria, explained the project to them, and handed out the corresponding information sheet. If a patient were willing to participate in the study, they were offered the option of either contacting the research team themselves or being contacted by her. In this subsequent contact, the researcher explained the study in detail and answered any possible doubts the patient might have. Then, if they expressed their wish to participate, a suitable date and place for the interview was arranged, according to the patient’s needs. The researcher facilitated their contact details in case the participant decided to change their mind and did not want to participate. On the day of the interview, if necessary, possible doubts were clarified and the interview began once the informed consent had been given and signed. Patients could carry out the interview either alone or accompanied by their relatives, according to their preferences.

### Interviews

The same member of the research team conducted all the interviews with the patients. A semi-structured guide (supplementary file 1) was developed for conducting the interviews with each patient, all of which were audiotaped and later transcribed. They began by referring to the experience of the patient’s WTD, mentioning that it is a common experience in patients with advanced disease, and that according to their physician or psychologist they had experienced this at some point in the trajectory of their condition. This was followed by showing interest in the patient’s own lived experience, inviting them to share it with the researcher. The researcher showed throughout the whole interview openness and interest to listen their detailed experience regardless what it was. Subsequent questions were flexible to the responses of the interviewee and helped the patient to go into more detail about their experience to derive their lived account. Finally, patients were asked about what was important when sharing this experience and what they considered to be helpful for patients who may feel the same as them.

### Data analysis

Data analysis was led by the same researcher who had conducted the patient interviews (AB) and used NVivo V.12 software for data management. During the data collection period, the researcher began to read the already conducted interviews and initiate the analysis process by identifying categories and codes in them. This initial analysis allowed for exploration and verification of the themes emerging from the patients’ accounts.

Once all the interviews with the patients had been conducted, the researcher continued the data analysis together with two experts in qualitative research (PL and MA). Each transcript was read in-depth, initially establishing a general or macro understanding of each patient lived experience. Subsequently a micro-thematic analysis was performed for each one, allowing the identification and coding of every relevant detail. This process was carried out for all patient accounts.

Throughout the analysis, we engaged in reflection and interpretation of both common and uncommon aspects identified. We continuously compared the common thread within these interpretations to discern the characteristic elements of this experience, without which the experience would cease to be what it is.

Weekly meetings were held to discuss the codes and categories that had been found during the data analysis, leading to the identification of the common characteristics of the experience.

The process of data collection ceased when the additional data did not lead to any new emerging themes and there were enough recurring patterns to illustrate the phenomenon [23].

A total of ten main subthemes were clustered during the analysis, which led to the study results (Table 1).

**Table 1:**
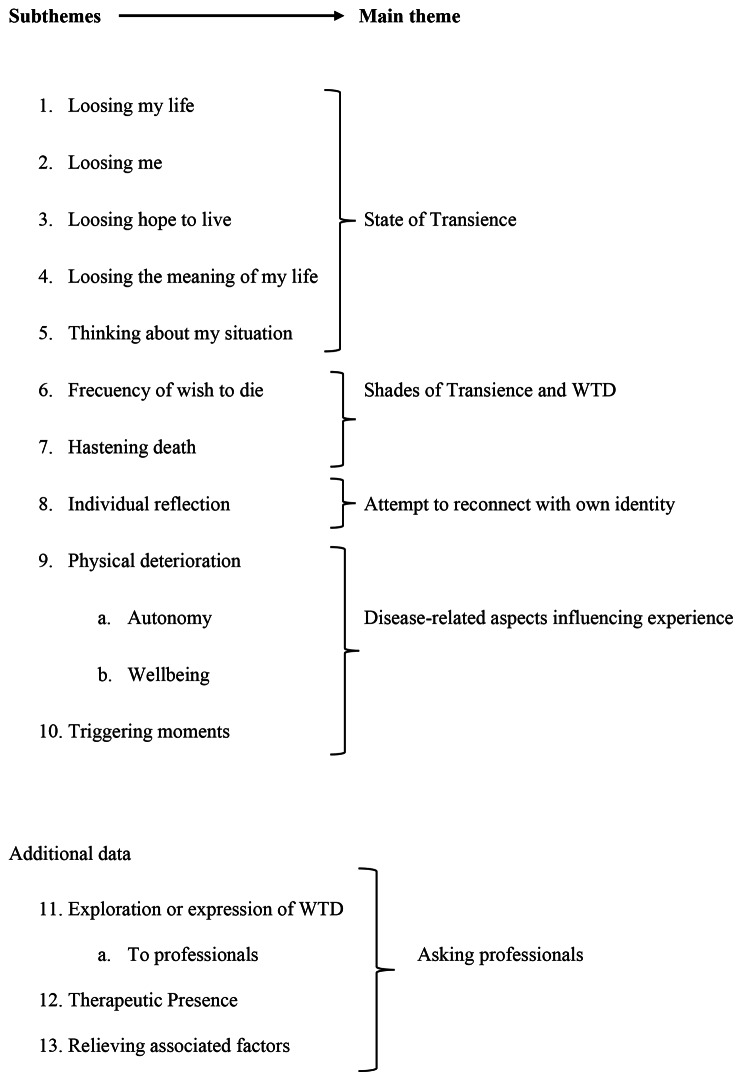
Clustering subthemes into main themes

## Results

### Sample

The 14 patients who had been invited to participate in the study (seven from the CUN, two from the HSJD, and five from OSI ORABA) accepted the invitation. Their sociodemographic characteristics are shown in Table 2. The interviews, with a mean duration of 40 min (range 25–60 min), were conducted either in the outpatient clinic (*n* = 2), at home (*n* = 4), or in the hospital setting (*n* = 8).

**Table 2:** Sociodemographic and clinical characteristics

Total patients (*N* = 14)	*N* (%)
Age (*n*, range)	71 (39–88)
Sex: Female	7 (50)
Marital status	
Married or with a partner	11 (79)
Widower	2 (14)
Single	1 (7)
Patients self-reported spirituality	
Yes	11 (79)
No	3 (21)
Disease	
Oncological disease	12 (86)
Other	2 (14)
Disease duration (number of years, range)	5 (0.4–16)

### Findings

Three common characteristics with respect to the experience of the WTD in the study participants: the state of transience, the attempt to reconnect with their own identity, and disease-related aspects that influence the lived experience. In addition, three subthemes containing patients’ recommendations for exploring and managing the WTD were identified.

#### Common characteristics of the experience of wish to die

##### Patients are in a state of transience

The patient with an advanced disease perceives their life threatened and initiates an introspective process, from which arises the experience of wish to die. This introspective exercise is carried out from a state of transience, defined as an ephemeral state of existence where things once considered permanent have changed [24]. Thus, the connection between the wish to die and transience is based on the perception of life’s potential finiteness. Thereafter, the patient reflects on the meaning of their own life by reviewing their life’s journey, and perceives the ephemerality of life itself. This reflection extends beyond the finiteness of their life. The patient contemplates their life within the overall framework of their values, questioning and even feeling uncertainties that sometimes they themselves are not able to specify or understand. The patient may even experience a sense of rootlessness because they have to cope with the unknown.

*This is when you start to rethink your life. I’m single, many times I find myself alone, I find myself useless, although, thank God, I have many friends, but that’s when you raise a lot of issues. (P.05)*

*Lately it has become more intense (the experience of WTD)… I see it (my future) very badly. Besides, now I am thinking about it, I don’t want to be like this, I don’t want to suffer, I want to leave, I’ve already lived. I want to leave without suffering. And I think about it and I say: “Well, what do I know how I’m going to end up? (P.01)*

*It’s just that I realize I can’t live a normal life. And yes… I don’t think it’s worth living just to suffer. After all, my life has been a great suffering, I have had good moments, but many of them have been bad (due to the disease) and you think: Is it worth living“(P.06).*

*For me it is a process. It is not to say: <<I am well and suddenly, from one day to the next, I want to commit suicide because I am not well>>. I don’t think so. At least not in my case. In my case it was to say: Damn, I don’t know about this, and there comes a moment and you say: “There’s no point in being like this”. There comes a moment, but after time and time you see that you have no solution. That’s when you say: “Is it worth it?” You start to think about those little things at that moment (P.05).*

In this situation, the patient experiences sadness and other painful feelings [24] such as demoralization or hopelessness. The attributes that emerge in this state are the patient’s fragility, the feeling of impermanence and irreversibility, and stasis. There is not so much in process, it is a sense of stasis [24], a limitation in their ability to move forward. This limitation renders the patient immobile in the face of the disease situation and causes a tunnel vision, which makes it difficult to envision a better future.

*Somehow, to get out of the way and say: “Let’s see, I’m going to be beating myself up for two, three, four, five, as long as the disease lasts, if there is no solution and my quality of life is going to be like this, I cannot ruin their lives.” (P.05)*.

*I do not do anything here, there is no hope for anything… what I have left is this… is to be bedridden and that’s it” (P.07)*.

Their experience of the disease and their situation becomes so important that they feel powerless and sometimes it is difficult to focus on what is really important to them.

*It is difficult… Being aware of your illness all day distracts you from other things that are very important. For example, my wife, we have been together for 50 years… we have done many things together, but I no longer feel capable of anything, I do not feel capable of taking her anywhere because I have no strength. So, I feel that this is an extension of a meaningless life (…) Those things we have enjoyed together are not possible now. (P.03)*

### Shades of transience and the different experiences of wish to die

The common aspects of transience have been described above, but nuances are also observed in some of the patients interviewed. On the one hand, the WTD experience reflects the current state of the person, not a concluded process. In other words, this state of transience is experienced at a specific moment. But this sensation that everything changes, that everything is ephemeral, is not constant and its intensity can oscillate depending on the patient’s moment. Therefore, the patient can move from that state of transience in various directions. The WTD experience might disappear if the situation improves; and it may reappear when the circumstances that triggered such experience in the patient occur again.

*I told her (my wife) that if this pain does not go away and is so strong and continuous, because most of the time it hurts for 24 h, it is not worth continuing to live with this pain. It would be preferable, as soon as possible, to end this suffering. (P. 02).*

*Let’s see… that day was the toughest day I have ever experienced WTD, but I had the idea of leaving (referring to WTD) before when I got out of there, from Barcelona, from that hospital, because I thought “Well, if I’m so bad - because I was really bad - I’ll just leave. (P.01).*

On the other hand, it is also observed that from that state of transience, where the patient is in a period of stasis experiencing painful feelings, some patients carry out a transition, understood as a process that involves a movement of the patient facing this situation, who moves on. The patient who carries out such a transition undergoes a process of becoming [25], arriving, in this particular case, at a way of being. This desire to die fills the patient’s daily life and could rarely be modified because they seem to have lost the ephemeral state that characterizes transience. It is observed that the time factor, specifically, that this state has been maintained for a long time, has a relevant effect on whether this state has become an attitude or a way of leading their everyday life.

*It’s not that I don’t want it, it’s that I’m asking for it, do you understand me? I don’t want to be suffering, what for? If I know what I have, the disease I have, that I have cancer and I know that I can’t go as far as I have to go. I have been like this for a long time, and I have made the most of it (the time I have lived), I have been with it for eight years, and I have gone through these times that I have had, sometimes bad times, life is like that, nothing more. (P.03)*

*I’m telling you, and the thing is that maybe, really, really, from the truth of my heart, maybe you don’t want to live. I know that I’m going to be in heaven more time than here, but there are times when I say: “I’d go to sleep and not wake up at dawn”. Lately I’ve got it into my head that no, I don’t know what I’m doing here. (P.09).*

*My daughter tells me that if I’m here it’s because I have to be. I say: “no, no, no. I don’t have to be here because I don’t, because sometimes I get bored, I feel like going to heaven. No, no, no, no, no, I see that here I don’t… No, I’m not, I’m not doing well. Lately I’ve been doing it badly. I have it stuck in my head there, going round and round in circles. I want to forget about it, but no, it’s there. (P.12)*

There is another nuance that sometimes appears in the WTD experience and that has to do with hastening death. Patients who report a more sporadic (less persistent) experience, of wish to die, sometimes mention the idea of doing something (e.g. asking for euthanasia) for hastening death. This thought is presented as an ideation, as something possible that could come true. These patients are in that period of transience in which the experience might not be constant or even reversible, so the way in which they live the idea of hastening death is hypothetical.

*I pray to God to take me now, to take me with him as soon as possible (P. 11).*

... *but of course, it’s not that easy. If I said “Look, I’ll let myself and that’s it”, but of course, what happens? Imagine that you do it and you have all this time ahead of you, like now (P. 01).*

Those patients who transmit a more persistent or continuous experience of desire to die, suggesting that they have already made the transition that has been termed ‘desire to die’, may or may not experience the desire to hasten death. In those cases, in which the actual idea of hastening death appears, it is presented as something intentional and more structured.

*If I could have had a final solution, which was nothing more than taking a pill and sleeping. I would have taken it. (P. 03).*

*I’m going to ask for euthanasia sooner rather than later. (P. 13).*

#### The patient’s attempt to reconnect with their own identity

In the state of transience, the patient performs an exercise of introspection in their attempt to reconnect with their own being. They reflect on the meaning of their own lives by looking back at them. As they want to feel a desire to live, they try to find aspects in their lives that might help them in this respect, but these are not enough. There is a sense of wanting to hold on to something from their own life when it is difficult due to the change in circumstances.

*I have taken all the paths that could give me satisfaction: work, my children and my grandchildren. All that is in order, but now the only one that I think is left over in this whole setup is me… So, I tell you that I don’t mind dying. I don’t mind because I think that my life, in general terms, has been satisfactory, good and, at the same time, I have achieved goals far superior to those achieved by my parents and my siblings. (P.03)*

#### Disease-related aspects that influence the lived experience of wish to die

When patients narrate their experiences, they refer to the impact that the disease, its symptoms, and possible complications have on their WTD experience.

### Progressive deterioration due to disease

Progressive deterioration appears as key in the experiences of the WTD. Study participants report a progressive deterioration of their functional status that causes losses in different aspects such as their perceived well-being, the meaning of their own lives, their sense of hope, the autonomy they enjoy, or the feeling of being in control of their own lives.

*I can’t do anything by myself… I need help with everything… my husband has to take care of me. Sometimes I can’t take things by myself… you feel a lot of helplessness. I see myself locked up. (P.04)*

The patients themselves experience their deterioration by comparing their current lives with their previous ones. The result of this comparison is negative as these losses become more evident due to the course of the disease.

*It’s that I have fatigue at all times… Right now, tiredness… Eating, drinking… it tires me… it’s difficult… and you start thinking about it over and over again…… my body is a decaying body. (P.08)*

### Influence of incidents and complications in the disease process

The complications of the disease experienced by the patient can sometimes become unbearable and cause exhaustion.

*That’s where I’ve gotten to because, well, there comes a time when you say you get treatment, it doesn’t work, you get another one, it doesn’t work either. You can’t stand the pain. You start, in some way, to have to rely on others… there are many times that I have been very lonely when I have needed something, then you start to ask yourself: “Is it worth it (to live) like this to be constantly beaten down, so that I don’t have enough quality (of life) to say: ‘Can I get by on my own’? At least a little bit, don’t crush people so much. For me the biggest problem is people being crushed. (P.05)*

*Boredom, too. The morning goes by easily because I don’t get up early. I say: what’s the point, but the afternoons are… Some days we go out for a walk, but I don’t feel well either, because I have been a very busy person, I’ve moved here, I’ve gone there, I have liked to go for a walk, to go into the stores to see things. And all that weighs me down a bit. I can’t do it anymore, although I know I have to make myself aware that I can’t do it, but I feel bad… And very bored. Boredom, and when the night comes, I can’t cope with my life of boredom and tiredness. A tremendous tiredness. I say to myself: but what am I doing to be this way, so tired? I go from here to the dining table and from taking a nap and from bed to here. I do nothing else because I can’t do anything else. (P.09)*

*Pain changes your life, your behavior… I keep telling my wife, “give me something, no matter what, but something, this is impossible, it is inviable, there is nobody who can stand it. (P. 02)*

In other cases, patients report that the new symptoms or complications that arise throughout the course of the disease result in a feeling of exhaustion and are perceived as unbearable.

*I am inside my body. My body is that of an idiot, a body at the end of life going away. The bottom line is that, life is not worth living because I have no chance of this ending up well. I’m getting doing worse and worse every day, and these last few months things have gotten a little ugly. (P.04)*

*I have always had a very bad time (referring to disease) with physical wear and tear, mental wear and tear, especially of the system, mental system. I get all these treatments, but I feel helpless… bad… and I told the doctor… to anyone… that what I desire is to die. (P.03)*

#### What do patients experiencing a wish to die ask professionals?

##### They ask for a context of trust and security to talk about their WTD experience

In sharing their stories and perceptions, patients emphasize the importance of adequate communication in the patient-professional relationship about the experience. They mention two essential aspects for such communication to take place.

First, they emphasize the need to feel in a *safe space* in order to be able to express the experience of WTD. They also stress the importance of having a *relationship of trust* with the person with whom they discuss the WTD.

*I talk about it with my mother and my wife. Also, with some friends… they know me well and know all my process… they understand me… For a person who doesn’t know me it would be more difficult, but for those who know me, those who know what life I have had… (P. 06)*.

In addition, they convey that the proactive exploration of the WTD carried out by the professional should be done at a time when the symptoms are not so acute, and when essential aspects such as a relationship of trust and a safe space are guaranteed.

*It’s not good that anyone comes and asks you. When the conversation comes up, you talk about it, but when you are calm, don’t let them come looking for it, what for? To cry? I have spent many hours crying, and it doesn’t do any good. (P. 02)*

*I haven’t talked much with the doctors about it. More with people I trust… my wife, my mother and my friends… it takes trust to talk about it… with the health professionals I have also felt understood, since they have been seeing how my disease is getting more complicated. (P. 06)*

*At that moment I would have thrown myself out… I would have thrown myself out of the window… when the pain goes away you see everything from another perspective, but at that moment, it was the only thing I wanted. (P.05)*

##### They expect a response from professionals regarding what they have shared

Patients who discuss their wish to die expect a certain response from healthcare professionals. After establishing the conditions necessary to talk about their wish to die, patients suggest how professionals might act when they share this experience. Patients discuss “being” and “doing”. “Being” refers to feeling heard, acknowledged and not judged when sharing their experience.

*I don’t know, listen to them, and see what they want. And take into account their opinion a bit, which is also their life. It is your body, you are the one who knows how you are. Doctors know, of course, but you’re suffering it (P. 01).*

*It seems to me that you are doing very well, at least with me (referring to symptom relief and presence). I have found such a good host (P. 03).*

“Doing” relates to what patients expect from health professionals in terms of actions and support during their experience. Patients seek either therapeutic adjustments that do not prolong their suffering and allow the disease to follow its natural course, the alleviation of suffering-causing factors, or, in some cases, the initiation of euthanasia procedures.

*I’ve thought about being in a place that can let you go. Takes good care of me, and doesn’t let you suffer. I always say that I want quality, not quantity. I don’t want a long and bad life, of pain and this, no, no. I want to be like now, even if it’s shorter (P. 01).*

*To me personally taking away the pain doesn’t mean that I do not desire to live. It is the pain that takes away my will to live (P. 06).*

*Heal me. It is black or white. Either you cure me or euthanasia. (P. 04).*

## Discussion

This study has identified common characteristics of the WTD in a number of patients, providing an in-depth look at the experience itself. In addition, insights into the desired approach of patients with such experience have emerged from the analysis of the interviews.

To the best of our knowledge, this is the first time that the concept of “a state of transience” is addressed in the WTD experience. This term refers to an ephemeral state of existence, where the patient experiences the finiteness of life, accompanied by negative feelings such as demoralization, hopelessness, or sadness [24]. During this period, the patient reflects on themselves and tries unsuccessfully to reconnect with their own selves, which leads them to a state of stasis in the face of the situation they are experiencing. Chochinov already described an attempt to reconnect with oneself in near-death patients, where the patient with a fractured personhood struggles to get to the core of their being [26].

In this state of transience, patients seek to cling to something, such as the life they had before the disease onset, but this is no longer possible [24]. This can create a state of brokenness, where the patient feels that they are no longer the person they used to be [26] which can lead them to the experience of the WTD.

A previous phenomenological research in cancer has mentioned the presence of “explicit finitude” where patients think about the limited lifespan and death [27]. Similarly, William et al. talk about the “existential experience” in cancer patients, where they become aware of their own mortality and death [28]. They describe that once they have experienced this awareness, frailty and the threat of illness become present in their lives. They suggest that acknowledging these existential concerns could encourage coping [28]. Patients with the WTD might attempt to reconnect with their own self in the coping process that results in a transition in the living experience of their illness [24, 28, 29]. In this respect, Ohnsorge suggests that the WTD may be part of a process of acceptance of the terminal situation [3].

Furthermore, patients from this study were aware that they had a life-threatening disease and it has been observed that the patient perceives the threat that the disease poses to their own integrity, which suggests a state of suffering [30]. This suffering might persist until the threat disappears or until the individual’s integrity can be restored in some way [30]. Therefore, that suffering patient might be trying to restore their integrity with the aim of making a transition, understood as a move on [25], towards a state of adaptation to the new scenario they are facing.

Previous research studies have described various processes of adaptation and transition in patients with advanced diseases [29, 31, 32]. However, in the case of patients expressing the WTD, a profile characterized by a state of transience is observed. In such state stagnation prevails, hindering the transition in motion [33] that could lead to future existential growth [34]. Furthermore, the results of this study suggest that some WTD patients experience a transition towards a process of becoming individuals who DTD. Likewise, it is observed that the patient in transience could also transition to a state where the WTD experience disappears [1]. Therefore, the present study suggests at least two directions in which the patient in a state of transience could move: to a referral of the WTD or to a state of being with the DTD. However, it is emphasized that while DTD implies a completed transition, in the case of WTD it is unknown whether the patient has undergone a transition. This is due to the inherent dynamics of WTD [17], which, combined with the frailty of patients with advanced disease, makes it difficult to determine whether the remission of the WTD is permanent.

Likewise, the feelings that characterize this state of transience such as demoralization, hopelessness or sadness [24] have been historically described as factors related to the experience of wish to die [2, 7, 9, 14, 33–35].

Progressive deterioration and complications arising from the disease are influential elements in the WTD experience. It is crucial, therefore, to identify how each patient perceives the ongoing deterioration associated with their disease. Coyle, in her phenomenological study, has highlighted the debilitating progression of the disease and the perception of constant losses in different aspects of the person, such as autonomy, dignity, self-worth, or meaning in life [36]. She also claims that such losses could be triggers for the experience of the WTD [36]. Furthermore, another research study has claimed that the influence of physical deterioration and losses as factors of existential suffering might cause an increase in requests for assisted suicide [37]. This study has also highlighted that, in this state in which the patient finds themselves, present and potential complications lead to exhaustion, which creates a state of constant suffering. Krikorian et al. have defined suffering as a multidimensional and dynamic experience of severe stress that arises in response to events that threaten the integrity of the person. In this state, regulatory processes, which would normally lead to adaptation, become insufficient and result in exhaustion [38].

The present investigation poses an innovative perspective on the construct of state of transience that encourages reflection on how healthcare professionals can recognize such state and approach patients in that period of their lives. To the best of our knowledge, this is the first study to ask patients how they believe this experience should be explored. This inquiry about what to do applies to patients with both a non-established (WTD) and a persistent experience (DTD), as the goal of health care professionals is always to provide personalized support, addressing their needs and seeking to alleviate their suffering.

The most recent studies on this matter have indicated that the proactive exploration of the WTD does not harm the patient, is considered important by the patient and does not increase the WTD in the patient [1, 6]. However, the participants in this study have emphasized the importance of this proactive screening being carried out in a context of safety and trust. These findings coincide with the Therapeutic Effectiveness model proposed by Chochinov, which advocates the creation of a safe space conducive to the therapeutic encounter [39]. This space is not limited exclusively to physical aspects; rather, it is a private, quiet space that guarantees confidentiality, allowing the patient to feel that it is their moment [39] and where healthcare professionals respect the patient´s pace.

On the other hand, patients have explained how their care needs are centered on “being” and “doing”. Being focuses on responding to the existential suffering experienced by the patient. Providing care centered on the patient’s existential sphere could facilitate transitions related to illness and the end of life [28]. To this end, it is essential to preserve the care tenor, ensuring that the tone of care the patient receives affirms the dignity of the individual.

This tenor of care translates into the “therapeutic presence” of the professional, and is considered a key intervention within the WTD experience [39]. The concept of therapeutic presence speaks of a state in which the health professional, from their own being, is completely immersed in the moment of encounter with the patient, encompassing physical, emotional, cognitive, spiritual, and relational aspects [40]. To achieve this, the professional must maintain contact with themselves while remaining receptive to the process, showing openness at the moment of the encounter. The intention is to be at the service of each patient’s lived process [39–41] without trying to avoid or change the patient’s WTD experience. The therapeutic presence is described, among other attributes, as being compassionate and empathic, respectful and nonjudgmental, authentic, fully present, and valuing the intrinsic worth of the patient [39].

Moreover, interventions designed to favor meaning and purpose have been shown to alleviate depression, demoralization, or the WTD in oncology patients with existential distress [34, 42]. Considering the results of the present study, interventions that promote the reconnection with oneself, as well as the affirmation of the perceived dignity, could be key elements in the care of patients experiencing the WTD. Among these interventions, research studies have suggested Dignity Therapy [43] and the Patient Dignity Question (PDQ) [44]. In the PDQ patients review their lives, which allows them to find the person they still are and to strengthen the perception of their own dignity [43, 45]. On the other hand, the PDQ can help the person to affirm their identity in the face of the disease they have, contributing to the professional’s recognition of the person they are caring for, which might lead to a humanized tone of care [44].

Some limitations to the study are noted. It intended to study the experience of patients with all types of advanced disease and in different situations, but the sample included few non-cancer patients, and all participants were supported by palliative care teams. For this reason, it should not be generalized to all WTD experiences. It might also be interesting to analyze experiences of greater and lesser intensity (from a more sporadic WTD to an established DTD) separately in an attempt to better characterize each one.

## Conclusion

This study delves into the lived experience of the WTD patient, identifying three fundamental characteristics that refer to the state of transience, the attempt to reconnect with oneself with the aim of recognizing oneself and disease-related aspects that influence the lived experience of the WTD. In addition, it reports on the needs of patients in terms of how healthcare professionals should approach and accompany them throughout this experience. Both therapeutic presence and interventions that promote the perception of dignity are presented as key elements in the care of patients with WTD.

### Electronic supplementary material

Below is the link to the electronic supplementary material.

Supplementary Material 1

## Data Availability

No datasets were generated or analysed during the current study.

## Abbreviations

WTD: Wish to Die
DTD: Desire to die
