# Supplementary material for: The state of transience, and its influence on the wish to die of advanced disease patients: insights from a qualitative phenomenological study
Source: BMC Palliat Care. 2024 Feb 26;23:57. doi: 10.1186/s12904-024-01380-z (PMC10895803; doi:10.1186/s12904-024-01380-z)
Supplement: Supplementary file 1 — Supplementary Material 1 [file 12904_2024_1380_MOESM1_ESM.docx]

**Supplementary file 1. Semi-structured interview guide.**

**1. Introduction and Opening Question:**

Some people with an illness sometimes feel that they wish to die, and as you know, we are conducting a study to better understand this feeling.

According to what your doctor/psychologist has told me, at some point you have felt that you wished to die. I would like to talk about it, but…

*… before we specifically discuss your experience of wish to die, I would like to know more about you and understand your experience of having an illness. Could you tell me about it?*

**2. Experience of Wish to Die:**

*… and now, could you tell me what your experience of wish to die is like?*

**2.b.Evolution over time:**

*Some people tell me that the experience of wish to die changes over time. How has your experience been?*

**3. Associated Factors:**

I am interested in factors/aspects/things that have affected or influenced your wish to die.

*In your case, what would you say has influenced you?*

**4. Communication of the experience:**

*I wonder who you have talked to about this experience of wish to die…*

*How was this conversation? How did you feel?*

**5. Hastening Death:**

When I talk to people who have the idea of wish to die, sometimes they tell me similar experiences to yours (mention something about frequency, evolution over time)...

... and sometimes they talk to me about wanting to do something to hasten their death (or for death to come sooner).

*What is your experience regarding this?*

**6. Interventions:**

Thinking about other patients who may feel the same as you…

*…What do you think could help them?*

**7. Before closing the conversation:**

*How has this interview been for you?*

*Is there any issue you would like to talk about that I have not asked you?*

**CONCLUSION:**

- Summarize what has been discussed (always positively) and ask: "Do you think I have understood it well?
- Everything you have mentioned is important; I would like to thank you for your participation in the study and for sharing your experience with me.
- Open door: in case you want to tell me something else, don't hesitate to contact me, and we can meet again.
